# Developing high throughput genotyped chromosome segment substitution lines based on population whole-genome re-sequencing in rice (*Oryza sativa *L.)

**DOI:** 10.1186/1471-2164-11-656

**Published:** 2010-11-24

**Authors:** Jianjun Xu, Qiang Zhao, Peina Du, Chenwu Xu, Baohe Wang, Qi Feng, Qiaoquan Liu, Shuzhu Tang, Minghong Gu, Bin Han, Guohua Liang

**Affiliations:** 1 Jiangsu Key Laboratory of Crop Genetics and Physiology/Key Laboratory of the Ministry of Education for Plant Functional Genomics, Yangzhou University, 88 Daxue Road, Yangzhou 225009, PR China; 2 National Center for Gene Research and Institute of Plant Physiology and Ecology, Shanghai Institutes of Biological Sciences, Chinese Academy of Sciences, Shanghai 200233, PR China; 3 Lixiahe Region Agricultural Research Institute of Jiangsu, 225007, Yangzhou, PR China

## Abstract

**Background:**

Genetic populations provide the basis for a wide range of genetic and genomic studies and have been widely used in genetic mapping, gene discovery and genomics-assisted breeding. Chromosome segment substitution lines (CSSLs) are the most powerful tools for the detection and precise mapping of quantitative trait loci (QTLs), for the analysis of complex traits in plant molecular genetics.

**Results:**

In this study, a wide population consisting of 128 CSSLs was developed, derived from the crossing and back-crossing of two sequenced rice cultivars: 9311, an elite indica cultivar as the recipient and Nipponbare, a japonica cultivar as the donor. First, a physical map of the 128 CSSLs was constructed on the basis of estimates of the lengths and locations of the substituted chromosome segments using 254 PCR-based molecular markers. From this map, the total size of the 142 substituted segments in the population was 882.2 Mb, was 2.37 times that of the rice genome. Second, every CSSL underwent high-throughput genotyping by whole-genome re-sequencing with a 0.13× genome sequence, and an ultrahigh-quality physical map was constructed. This sequencing-based physical map indicated that 117 new segments were detected; almost all were shorter than 3 Mb and were not apparent in the molecular marker map. Furthermore, relative to the molecular marker-based map, the sequencing-based map yielded more precise recombination breakpoint determination and greater accuracy of the lengths of the substituted segments, and provided more accurate background information. Third, using the 128 CSSLs combined with the bin-map converted from the sequencing-based physical map, a multiple linear regression QTL analysis mapped nine QTLs, which explained 89.50% of the phenotypic variance for culm length. A large-effect QTL was located in a 791,655 bp region that contained the rice 'green revolution' gene.

**Conclusions:**

The present results demonstrated that high throughput genotyped CSSLs combine the advantages of an ultrahigh-quality physical map with high mapping accuracy, thus being of great potential value for gene discovery and genetic mapping. These CSSLs may provide powerful tools for future whole genome large-scale gene discovery in rice and offer foundations enabling the development of superior rice varieties.

## Background

Genetic populations provide the basis for a wide range of genetic and genomic studies and many types of populations have been used for genetic mapping, gene discovery and genomics-assisted breeding. Construction and utilization of a suitable genetic population is pivotal for fine mapping and map-based cloning of quantitative trait genes, the most general strategy in plant molecular genetics and genomics.

Over the past few decades, many different types of populations have been used to identify and map quantitative trait loci (QTLs). A large number of these have been mapped to putative genomic regions, but few have been fine-mapped or cloned [1-26]. Researchers have attributed this to several factors including insufficient population size, unstable statistical thresholds for detecting putative loci, using a minimal number of molecular markers for analyses, and the low heritability of target traits [27]. However, the main reason for a lack of fine-mapping or cloning is the limitations of the populations used.

Early temporary primary mapping populations such as the F_2:3 _and BC_1 _families have been used for genetic analysis and a number of QTLs with relatively large effects have been detected [1-7]. However, these types of populations are difficult to maintain and trials cannot be repeated as the same genetic composition can only be used once [28]. Therefore, it is difficult to produce convincing mapping results. To facilitate the genetic analysis of complex traits, some permanent primary mapping populations such as doubled haploid (DH) and recombinant inbred lines (RILs) have been developed. However, whilst these populations can be utilized in genetic and genomic studies, they are not adequate for further analysis such as fine mapping and characterization of target QTLs [29].

Advanced backcross populations have been developed and used, with near-isogenic lines (NILs) being the most representative type. NILs have distinct advantages for QTL identification; genetic background noise can be eliminated and a QTL can be visualized as a single Mendelian factor. Each NIL carries either one or more donor segments in the near-isogenic background of the recurrent parent, which reduces the effects of interference from the genetic background. Several QTLs have been fine-mapped or cloned on the basis of the NILs [14,15,17-24]. However, development is laborious and time-consuming, preventing many researchers from performing map-based cloning of QTLs [30].

Doi *et al. *and Kubo *et al. *suggested that the development of chromosome segment substitution lines (CSSLs) was a viable alternative for resolving issues that emerged during efforts to achieve precise mapping of QTLs [31,32]. CSSLs are a series of NILs in which the substituted segments of the wide population contain the entire information of the donor, while each CSSL carries one or more donor chromosome segments in the genetic background of the recipient. The main characteristic of CSSLs is that the substituted segments of each CSSL are stable. As a result, CSSLs are useful for genetic studies in terms of the detection and fine mapping of QTLs for genome-wide target traits, and for studying the interactions between QTLs. In addition, secondary F_2 _populations can be derived from a further back-cross between a selected CSSL and the recurrent parent, which can then be used for the fine mapping and positional cloning of interesting QTLs [33,34]. To date, several CSSLs in rice have been developed, and many QTLs for traits of agronomic importance have been detected in this way [30-32,35-39]. These achievements have undoubtedly enhanced the understanding of complex traits and promoted plant genomic studies.

Previously reported CSSLs were selected via marker-assisted selection (MAS) and genotyped using limited markers. It is suggested that the substituted segments from the donor were discovered exactly and the genetic background of each CSSL was detected accurately, as there was a limited number of molecular markers and double-crossovers were always present. Next-generation sequencing technology provides the capacity for parallel sequencing of genomes and the development of a sequencing-based high-throughput genotyping method that combines the advantages of ultrahigh-dense marker coverage and eliminates the likelihood of overlooking double-crossovers. The technology ensures high mapping accuracy and resolution, and more comparable genome and genetic maps among the mapping populations [40]. Since 2009, two rice RILs have been accurately genotyped using high-throughput techniques based on whole-genome re-sequencing and ultrahigh-density linkage maps were constructed for QTL mapping [40,41].

In this study, a broad population that consisted of 128 CSSLs was developed. The population was derived from a cross between two sequenced rice cultivars: 9311, an elite restorer indica cultivar as the recipient, and Nipponbare, a japonica cultivar as the donor. To identify the genetic background and exact length of the substituted segments, and subsequently enhance the accuracy of the QTL mapping, the CSSLs were subjected to high-throughput genotyping by whole-genome re-sequencing with 0.13× genome sequence per line, and an ultrahigh-quality physical map was constructed. Using the bin map converted from the ultrahigh-quality physical map associated with the culm length (CL) of the 128 CSSLs, the QTL analysis mapped nine QTLs, explaining 89.50% of the phenotypic variance for CL. A QTL of large effect was located in a 791,655 bp region that contained the rice 'green revolution' gene. The other QTLs were mapped to their specific intervals on rice chromosomes. This research will facilitate fine mapping and cloning of quantitative trait genes, providing foundations enabling the development of superior rice varieties. Furthermore, it will become possible to illustrate the genetic mechanisms of complex traits in plant functional genomics.

## Results

### Polymorphisms detected by SSR and insertion/deletion (InDel) markers between the two parents

**Figure 1:**
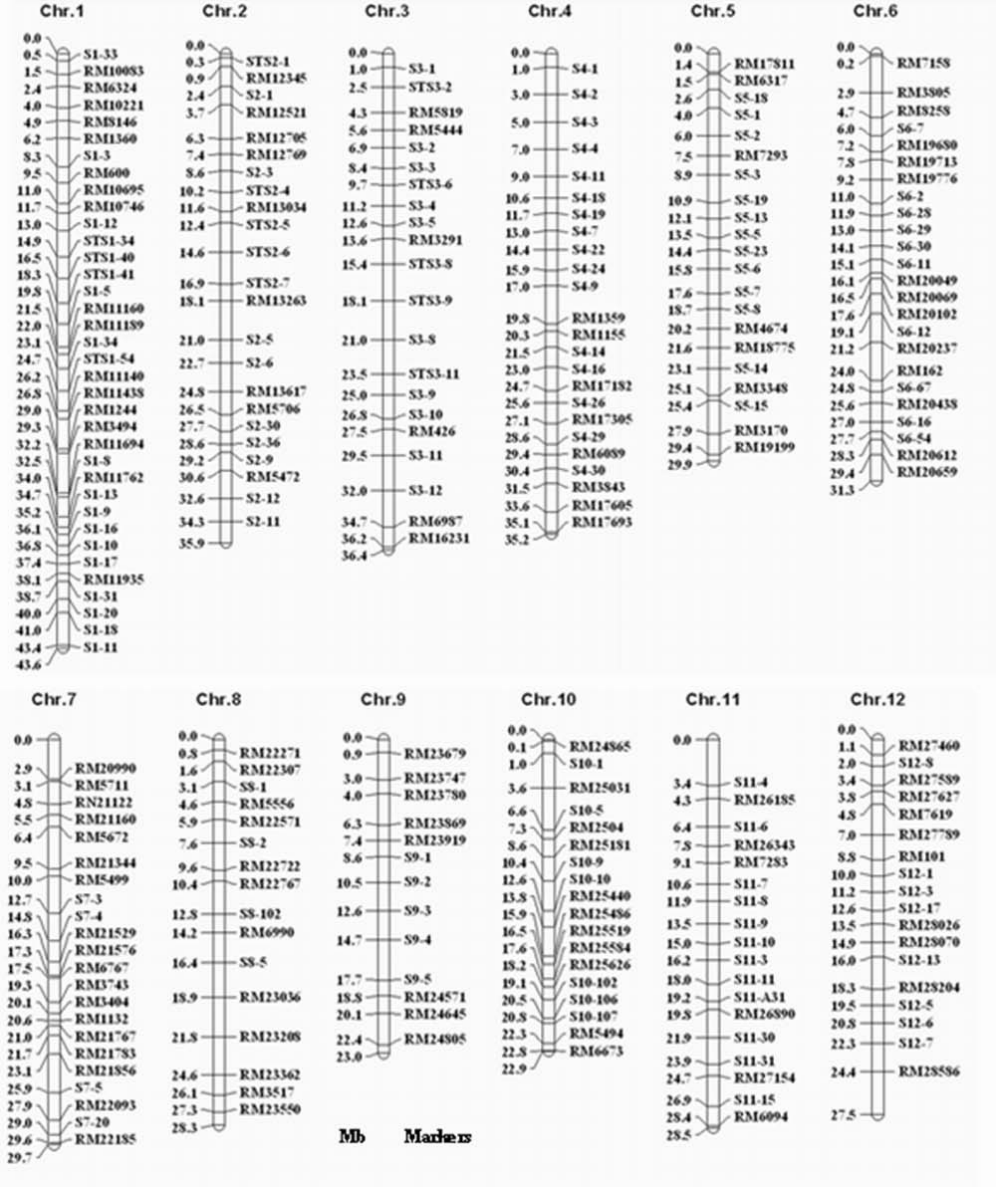
**The locations of the polymorphic markers in the rice physical map**.

A total of 739 markers were used in this study to survey the polymorphisms between 9311 and Nipponbare (Table 1). Of these, 460 simple sequence repeat (SSR) markers were selected and information regarding these was downloaded http://www.gramene.org, 279 insertion/deletion (InDel) markers were developed using Primer Premier 5.0 software according to the publicly available rice genome sequence comparisons that have been prepared for Nipponbare and 9311 http://rgp.dna.affrc.go.jp. Of the 739 markers, 254 (34.4%) displayed polymorphisms between 9311 and Nipponbare. The average size of the interval between two polymorphic markers was 1.46 Mb on the rice physical map (Figure 1). The polymorphic markers were utilized further for MAS in the process of the development of CSSLs and genotyping the 128 CSSLs.

**Table 1:** Summary of the markers used to develop the CSSLs.

Chromosome	Markers			Polymorphic markers		
	
	SSR	InDel	Total	Number	Percentage	Density (Mb)
1	44	43	87	36	41.4	1.21
2	36	20	56	23	41.1	1.56
3	52	12	64	21	32.8	1.73
4	43	30	73	24	32.9	1.47
5	48	30	78	21	26.9	1.42
6	40	30	70	24	34.3	1.30
7	40	20	60	22	36.7	1.35
8	43	20	63	16	25.4	1.77
9	26	5	31	13	41.9	1.77
10	35	20	55	18	32.7	1.27
11	19	31	50	18	36.0	1.58
12	34	18	52	18	34.6	1.53
Total	460	279	739	254	34.4	1.46

### CSSL development

**Figure 2:**
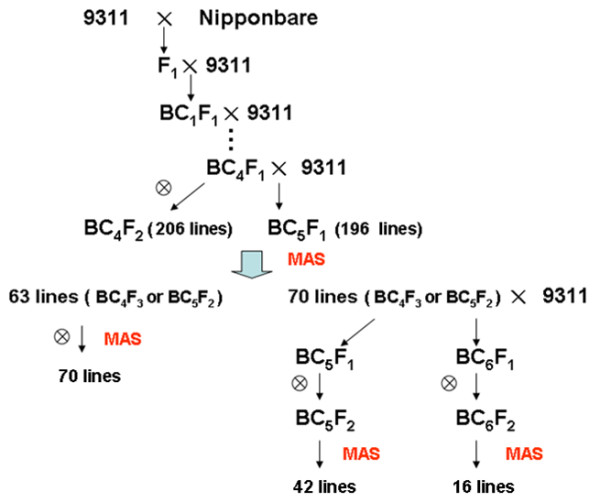
**Flowchart of the development of CSSLs in the present study**. MAS: marker-assisted selection.

The CSSL development procedure is summarized in Figure 2. The F_1 _plants were generated from 9311 'female' and Nipponbare 'male' parents. The F_1 _plants were back-crossed to 9311 to produce the BC_1_F_1 _generation. These BC_1_F_1 _plants were back-crossed with 9311 to produce BC_2_F_1_. Using the same method, 206 BC_4_F_1 _individuals were obtained. These BC_4_F_1 _plants were back-crossed with 9311 to produce BC_5_F_1_, which contained 196 lines, and were self-crossed to produce 206 BC_4_F_2 _lines. MAS with a whole-genome survey of 206 BC_4_F_2 _and 196 BC_5_F_1 _individuals, which were selected at random by taking one from each line, identified 133 plants in which the majority of genomic regions were homozygous for 9311 alleles; one to five heterozygous substituted segments from Nipponbare were the exception. Sixty-three plants that had one or two substituted segments were self-crossed to produce the BC_4_F_3 _and BC_5_F_2 _lines. Subsequently, 20 plants from each line were genotyped with the polymorphic markers on the target-substituted segments to select plants that had one or two homozygous substituted segments from Nipponbare; seventy plants were obtained. The remaining 70 plants, which had three to five substituted segments, were back-crossed with 9311 to produce the BC_5_F_1 _and BC_6_F_1 _lines, and these plants were self-crossed to produce BC_5_F_2 _and BC_6_F_2 _lines, respectively. Twenty plants from each line were genotyped on the target region to select plants that had between one and three homozygous substituted segments from Nipponbare; fifty-eight plants were obtained. The one hundred and twenty-eight plants that were obtained using these techniques were self-crossed to produce 128 CSSLs.

### Constructing the physical map of the CSSLs

**Figure 3:**
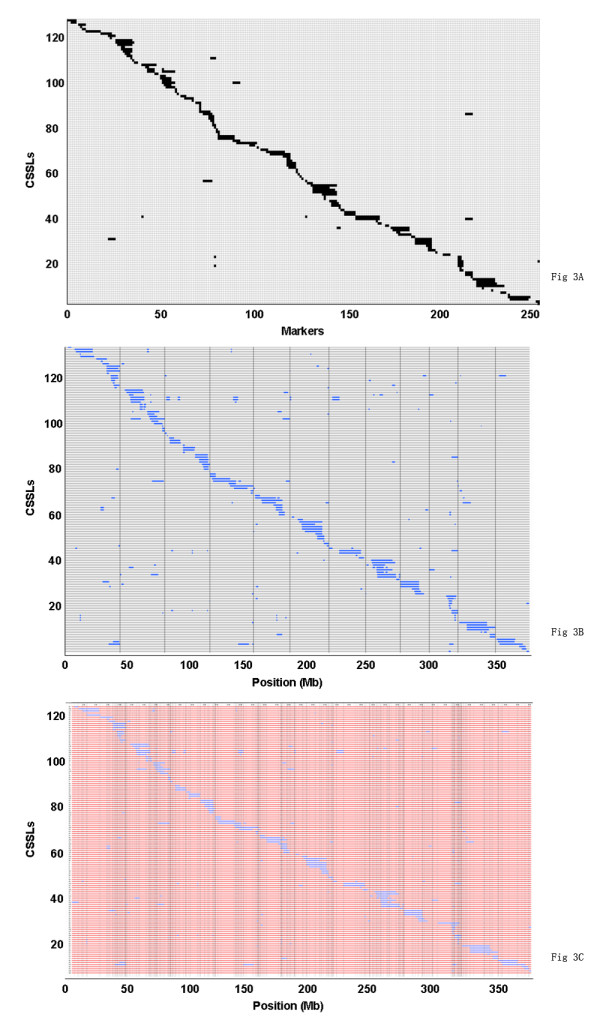
**The physical map and bin-map of the 128 chromosome segment substitution lines (CSSLs)**. **(A) **The physical map of the CSSLs was constructed with molecular markers. Each row represented a CSSL and each column represented a molecular marker locus. The black areas indicate regions that were homozygous for Nipponbare alleles; the white areas indicate regions homozygous for 9311 alleles. **(B) **The physical map of the CSSLs constructed by whole-genome resequencing. The blue areas indicate regions that are homozygous for Nipponbare alleles; the white areas indicate regions that are homozygous for 9311 alleles. **(C) **Bin-map of the CSSLs. The blue areas indicate regions that are homozygous for Nipponbare alleles; the red areas indicate regions homozygous for 9311 alleles.

On the basis of the physical locations and genotypes of the 254 molecular markers in the 128 CSSLs, the lengths and locations of the substituted chromosome segments were estimated. Using the results of the estimation, a physical map of the 128 CSSLs was constructed (Figure 3A).

When a 36-mer read of a CSSL was aligned to a region where a SNP was detected between the two parents, the genotype of the CSSL was assigned to this nucleotide position. Using the quality score of each SNP base as a filter, a total of 7.68 million high quality SNPs were detected. Therefore, every CSSL had approximately 60,000 SNPs (range, 3,576-180,935). Every SNP originated from Nipponbare or 9311. The average SNP density of the CSSLs was 0.16 SNPs/kb, or one SNP every 6.3 kb. On the basis of the physical locations and genotypes of these SNPs, each CSSL was genotyped, and a physical map of the 128 CSSLs was constructed (Figure 3B).

### Number, length and distribution of substituted chromosome segments in CSSLs

**Figure 4:**
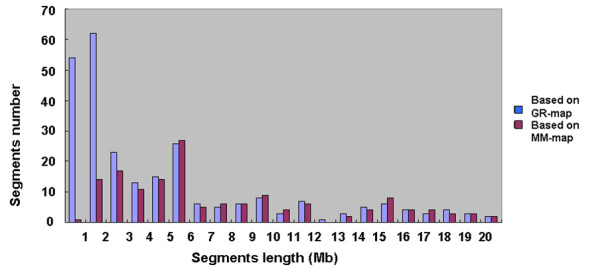
**Distribution of the length of the substituted chromosome segments in the 128 CSSLs**. MM-map: based on the physical map constructed with molecular markers; GR-map: based on the physical map constructed by whole-genome resequencing.

From the physical map constructed with molecular markers, the 128 CSSLs carried 142 substituted chromosome segments and each CSSL contained between one and three substituted segments from the donor in the genetic background of 9311. Of these, 115 CSSLs carried one substituted segment, 12 carried two, and one carried three substituted segments (Table 2). The length of substituted chromosome segments in the 128 CSSLs ranged from 0.65 Mb to 22.3 Mb, with an average of 6.21 Mb. Seventy-six segments were shorter than 6.0 Mb and 22 were longer than 15.0 Mb (Figure 4). The distribution of the segments along the chromosomes was not random. Different introgressed frequencies existed among the 12 chromosomes: of the 142 substituted segments, 20 existed on chromosome 1, while there were only six on chromosomes 7, 8 and 12 (Table 3).

**Table 2:** Segments carried by the CSSLs.

Segments carried	Number of CSSLs	
	
	MM-map	GR-map
1	115	54
2	12	41
3	1	18
4	0	8
5	0	5
6	0	2
Total	128	128

MM-map: based on the physical map constructed with molecular markers; GR-map: based on the physical map constructed by whole-genome resequencing.

**Table 3:** Distribution of substituted chromosome segments along chromosomes in the CSSLs.

Chromosome	Number of segments	
	
	MM-map	GR-map
1	20	32
2	18	35
3	19	32
4	10	20
5	12	25
6	18	23
7	6	9
8	6	23
9	7	11
10	13	25
11	7	16
12	6	8
Total	142	259

MM-map: based on the physical map constructed with molecular markers; GR-map: based on the physical map constructed by whole-genome resequencing.

The sequencing-based physical map indicated that the 128 CSSLs carried 259 substituted chromosome segments and each CSSL contained between one and six substituted segments from the donor in the genetic background of 9311. Of these, 54 CSSLs carried one substituted segment, 41 carried two, 18 carried three, eight carried four, five carried five, and two carried six (Table 2). The length of substituted chromosome segments in the 128 CSSLs ranged from 175 kb to 23.4 Mb, with an average of 4.91 Mb. Overall, 167 segments were shorter than 5.0 Mb and 22 of them were longer than 15.0 Mb. There were 117 'new' segments detected with whole genome re-sequencing that were not apparent in the molecular marker map; each of these newly revealed segments was shorter than 3 Mb (Figure 4). There were different introgressed frequencies among the 12 chromosomes: of the 259 substituted segments, 35 existed on chromosome 2 while there were only eight on chromosome 12 (Table 3).

### Genome coverage of substituted segments in the CSSLs

The physical map constructed with molecular markers indicated that the total length of substituted segments in the CSSL population is 882.2 Mb, which is 2.37 times the total length of the rice genome. The average number of substitution segments per chromosome was 12.5, ranging from six on chromosomes 7, 8, and 12 to 20 on chromosome 1. The average length of substituted segments per chromosome was 73.5 Mb, ranging from 41.3 Mb on chromosome 12 to 108.2 Mb on chromosome 6. The average rate of coverage of substituted segments per chromosome was 91.7%, although it varied from 66.7% on chromosome 10 to 100% on chromosomes 4, 5 and 11 (Table 4).

**Table 4:** Chromosome coverage of substituted segments in the CSSLs.

Chr	Length		Times		Coverage length		Coverage rate	
	
	MM-map	GR-map	MM-map	GR-map	MM-map	GR-map	MM-map	GR-map
1	104.16	157.27	2.39	3.49	42.39	41.23	97.2%	91.5%
2	99.90	166.78	2.78	4.53	32.78	35.24	91.3%	95.7%
3	83.20	114.56	2.29	3.07	32.93	35.30	90.5%	94.8%
4	66.00	108.95	1.87	3.04	35.20	35.86	100.0%	100.0%
5	72.61	122.71	2.44	4.08	29.90	29.86	100.0%	99.4%
6	108.25	106.60	3.47	3.32	30.00	29.56	95.8%	92.0%
7	55.35	59.31	1.86	1.95	27.00	28.92	90.9%	95.3%
8	56.60	125.19	2.00	4.39	24.76	27.62	87.5%	96.8%
9	65.46	73.09	2.84	3.07	21.23	22.98	92.3%	96.4%
10	47.07	65.41	2.06	2.76	15.27	14.05	66.7%	59.4%
11	82.33	104.22	2.89	3.38	28.50	29.42	100.0%	95.4%
12	41.25	66.40	1.50	2.39	21.39	26.36	77.8%	95.0%
Genome	882.18	1270.48	2.37	3.32	341.40	356.41	91.7%	93.3%

The sequencing-based physical map indicated that the total length of the substituted segments in the CSSL population is 1270.5 Mb, which is 3.3 times that of the rice genome. The average number of substitution segments per chromosome was 21.6, ranging from eight on chromosome 12 to 35 on chromosome 2. The average length of the substituted segments per chromosome was 105.9 Mb, ranging from 59.3 Mb on chromosome 7 to 166.8 Mb on chromosome 2. The average rate of coverage of substituted segments per chromosome was 93.3%, ranging from 59.4% on chromosome 10 to 100% on chromosome 4 (Table 4).

### Constructing the bin map and mapping the QTLs for CL

**Figure 5:**
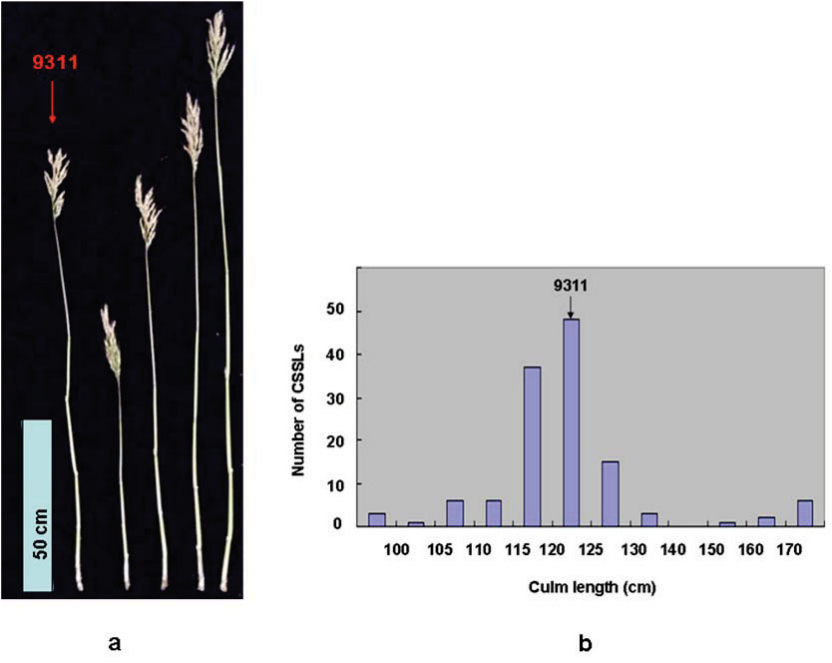
**CL of CSSLs under natural field conditions**. **(a) **The CL phenotypes in 9311 and CSSLs. Scale bar, 50 cm. **(b) **Distribution of CLs in 128 CSSLs under natural field conditions.

To evaluate the potential advantages of the CSSLs for QTL detection, phenotypic variations of the CL were observed in 128 CSSLs. CL ranged from 96.30 to 179.67 cm (Figure 5). The CL valus were 124.05 ± 0.12 cm in 9311 and 98.02 ± 0.09 cm in Nipponbare.

To conduct QTL analyses, the sequencing-based physical map was converted into a skeleton bin map. A total of 401 bins (defined as *x*_1 _to *x*_401_) were obtained for the 128 CSSLs. The average physical length of the recombination bins was 889,652 bp (range, 13,213-10,654,035 bp; Figure 3C). This bin map was used for mapping QTLs that control CL.

QTL analysis of the 128 CSSLs mapped nine QTLs: *qCL1-1, qCL1-2, qCL1-3, qCL1-4, qCL3-1, qCL3-2, qCL5-1, qCL6-1 *and *qCL8-1*, which were located in *x*_33 _on chromosome 1, *x*_34 _on chromosome 1, *x*_35 _on chromosome 1, *x*_36 _on chromosome 1, *x*_150 _on chromosome 3, *x*_152 _on chromosome 3, *x*_203 _on chromosome 5, *x*_227 _on chromosome 6 and *x*_278 _on chromosome 8, respectively. These QTLs explained 2.79%, 1.63%, 3.28%, 70.72%, 1.49%, 2.14%, 1.84%, 1.30% and 4.31% of the phenotypic variance of CL, respectively (Table 5). The QTL with the largest effect was mapped to *x*_36 _and occupied the physical position of 39,868,630 bp to 40,66,285 bp, which comprises a 791,655-bp region on chromosome 1 that contains the semi-dwarf gene *sd1 *[26].

**Table 5:** QTLs mapped for CL in rice.

No.	QTLs	Bins	**Chr**.	Interval	Interval size	Partial R-Square	Model R-Square	F Value
1	*qCL1-1*	x33	1	38016171-39172107	1155936	2.79%	2.79%	15.71
2	*qCL1-2*	x34	1	39172107-39372177	200070	1.63%	4.42%	12.92
3	*qCL1-3*	x35	1	39372177-39868630	496453	3.28%	7.70%	21.55
4	*qCL1-4*	x36	1	39868630-40660285	791655	70.72%	78.42%	306.76
5	*qCL3-1*	x150	3	35514007-35786915	272908	1.49%	79.91%	12.99
6	*qCL3-2*	x152	3	36109587-36127677	18090	2.14%	82.05%	21.8
7	*qCL5-1*	x203	5	18561151-18983458	422307	1.84%	83.89%	13.27
8	*qCL6-1*	x227	6	1-1171547	1171546	1.30%	85.19%	14.73
9	*qCL8-1*	x278	8	2797908-3336084	538176	4.31%	89.50%	21.77

## Discussion

### High-throughput genotyping of CSSLs is time- and cost-effective, resulting in highly reliable information regarding substituted segments

Molecular markers have been used successfully in genotyping assays for developing mapping populations and map-based cloning of genes in rice [42,43]. In this study, CSSLs were developed via MAS and genotyped using 254 markers. The process of collecting, designing, screening and amplifing using PCR, and scoring on agarose gels, took more than three years. Subsequently, the CSSLs were genotyped using high-throughput sequencing, and an ultrahigh-quality physical map based on whole-genome re-sequencing was constructed. This is the first study to use this method for genotyping CSSLs in rice, and remarkably, the entire process took only seven weeks. The sequencing-based high-throughput method is significantly more time-efficient and cost-effective and less laborious than the conventional PCR-based genotyping approach.

**Figure 6:**
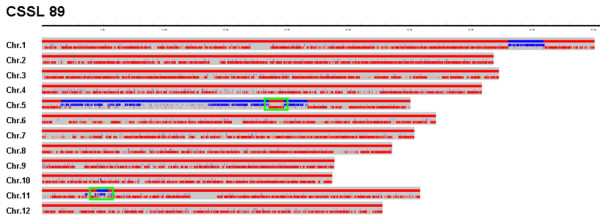
**Recombination map of CSSL 89**. Red lines: homozygous 9311 genotype; blue lines: homozygous Nipponbare genotype; green panes: double-crossovers.

Of most importance, the sequencing-based high-throughput method provides more information that is more accurate than the conventional approach. In this study, an average coverage of 6.3 kb per SNP was obtained, or 1 Mb per 159 SNPs for the CSSLs, a considerably better resolution than the 1.46 Mb per marker obtained with PCR-based markers. Therefore, the new method improved the resolution of recombination breakpoints 236-fold, and almost eliminated the likelihood of missing double-crossovers in the mapping population. As illustrated in Figure 6, two double-crossovers in CSSL 89 were detected by the sequencing-based method that had not been identified by the PCR-based method owing to limited marker density. Ebitani *et al. *constructed and genotyped CSSLs derived from a cross of 'Kasalath' and 'Koshihikari' using 129 RFLP markers [30]; 140 SSR and 5 EST markers were used by Takai *et al. *[37], and Zhu *et al. *used 132 SSR markers in their study [39]. Hence, the 254 markers used here represented a higher density than those used in the aforementioned reports. Nevertheless, employing the new sequencing-based approach, 117 segments, each shorter than 3 Mb, were identified that had not been revealed by the conventional PCR-based approach. The final analysis demonstrated that CSSLs derived from advanced backcross and self-cross lines contained a large number of double-crossovers rather than molecular markers. The sequencing-based method is perhaps one of the most suitable approaches to genotyping mapping populaions that provide an accurate foundation for QTL mapping.

### CSSLs derived from two sequenced cultivars can simplify map-based cloning of QTLs

The two subspecies of rice in Asia, indica and japonica, differ greatly in terms of their agronomic traits and have a strong potential for heterosis. A number of mapping populations derived from crosses between indica and japonica rice have been constructed for use in in-depth analyses of the genetic variations between the two subspecies [30,32,37,39,40]. The construction and utilization of the CSSLs derived from 9311 and Nipponbare, which are the typical varieties of indica and japonica that have undergone whole-genome sequencing previously, are highly consequential. First, the genomic sequencing information that has been generated has facilitated the search for high-density molecular markers for the fine mapping of QTLs for target traits. Second, once the location of a target QTL has been targeted to a certain region that contains a number of predicted genes, sequence analysis using the existed sequence information can narrow the search for candidate genes and allow them to be detected efficiently and quickly. Therefore, the use of this CSSL population, combined with the application of modern bio-informatics, can simplify the process of map-based cloning of QTLs of interest.

### The CSSLs constructed a platform for QTL mapping

CSSLs can be used for detecting and fine mapping of QTLs as a single Mendelian factor by blocking background genetic noise, which simplifies the process of data analysis and increases the accuracy of the results. To date, several sets of CSSLs have been developed and used for the mapping of QTLs [30,31,36-39]. However, the CSSLs described in the present study were developed via MAS and genotyped using limited markers, for which no accurate detection previously existed. This resulted in some double-crossovers being undetected, which was confirmed in the present study. Therefore, some QTLs may not have been detected, and others were mapped to the wrong region. In this study, we developed 128 CSSLs in the genetic background of rice cultivar 9311. Using these lines, combined with the sequencing-based bin-map, nine QTLs were mapped which explained 89.50% of the phenotypic variance for CL in these specific intervals on rice chromosomes. More importantly, a QTL with a large effect was found to be located in a 791,655-bp region that contained the rice ''green revolution'' gene. We believe that this level of detection would not be possible using other populations.

### The CSSLs constructed a platform for rice breeding

Plant breeding combines art and science to improve the genetic basis of new varieties of crops with increased productivity and quality [36]. Traditional breeding was predominantly based on phenotypic assays. Plant breeding systems have entered a molecular breeding era, where the process of molecular marker-assisted selection is used in plant breeding programs to combine phenotype and genotype. This approach shortens the breeding period and improves efficiency, and overcomes the traditional shortcoming of low accuracy with broad applications. However, thus far the method targets the QTLs of only one or a very few traits for genetic improvement. CSSLs selected at the level of the whole genome and multi-trait breeding objectives in areas such as the expansion of multiple targets have been pivotal in improving the properties of plants while leading the way in the ongoing technological innovation of plant breeding.

The elite indica cultivar 9311, used as the recipient in this study, has been planted on a large scale as an excellent variety, and has been widely used as a parent of super hybrid rice in China with a good grain shape, high eating quality, high yield and multi-resistance to disease. Some elite CSSLs in this wide population have a similar genetic background to 9311, but their comprehensive characteristics were better than 9311 in different environmental conditions (data not shown). Therefore, they could be used to create new varieties with direct marketing applications, and as a parent to create new hybrids. Furthermore, there are favorable alleles at the loci of interest that can be combined through MAS and lead to the production of superior rice varieties.

## Conclusions

We have successfully developed a wide population that contains 128 CSSLs, which were used to construct a platform for QTL mapping, cloning and marker-assisted breeding in rice. Each line was genotyped and a high-quality physical map of ultrahigh-density SNPs based on whole-genome re-sequencing data was constructed. Information related to substituted segments and the background of each line was considerably more accurate in this map than in a comparison map constructed using 254 PCR-based markers. The CSSLs described in this study are powerful tools for large-scale gene discovery and could have a significant impact on the future of the functional genomics of rice.

## Methods

### Plant materials

Two sequenced rice cultivars, 9311 and Nipponbare, were used to develop CSSLs. The elite indica cultivar 9311 was used as the recipient. Nipponbare, a japonica cultivar, was used as the donor.

### DNA extraction and molecular marker analysis

Genomic DNA was extracted from fresh-frozen leaves of each individual using the CTAB method as described by Rogers and Bendich [44]. The extracted DNA was dissolved in ddH_2_O. DNA amplification was performed by PCR with the following parameters: an initial cycle of 5 min at 95°C; 33 cycles of 30 s at 94°C, 30 s at 55°C, 40 s at 72°C; and a final cycle of 10 min at 72°C. Reactions were carried out in 96-well PCR plates in 25 μL volumes containing 1 μmol/L of each primer, 200 μmol/L of dNTPs, 5 ng of DNA template, 2 mmol/L MgCl_2_, 2.5 μL 10× buffer (supplied by Sheng-gong Inc. with Taq polymerase) and 1 U of Taq polymerase. Amplification products were analyzed on 3.5% agarose gels stained with ethidium bromide and photographed using a UVP system.

### Estimating of the length of substituted chromosome segments in CSSLs using molecular markers

The length of substituted chromosome segments in CSSLs was estimated on the basis of graphical genotypes [36,39]. A chromosome segment flanked by two markers of donor type (DD) was considered to have a 100% donor type; a chromosome segment flanked by two markers of recipient type (RR) was considered to be 0% donor type; a chromosome segment flanked by one marker of donor type and one marker of recipient type (DR) was considered to be 50% donor type. Therefore, the length of DD plus the length of two half DR was considered to be the estimated length of a substituted chromosome segment.

### High-throughput genotyping using whole-genome re-sequencing

A high-throughput method was used for genotyping the wide population of CSSLs utilizing whole-genome resequencing data generated by the Illumina Genome Analyzer IIx. As shown in Figure 2, CSSLs were developed from a cross between two sequenced rice cultivars 9311 and Nipponbare.

The genome sequence of Nipponbare has been reported (International Rice Genome Sequencing Project 2005) and was treated as the rice reference sequence. The 9311 genome used in this study was not the same as that reported previously [45]. Therefore, the genome was re-sequenced using the Illumina GAIIx for 2 × 76 bp paired-end sequencing in one lane, and yielded approximately 10× raw sequence. The 76-bp paired-end reads were mapped to the rice reference genome (IRGSP 4.0, http://rgp.dna.affrc.go.jp/IRGSP/Build4/build4.html) using Ssaha2 software v2.3 http://www.sanger.ac.uk/Software/analysis/SSAHA2/. Aligned reads were left with a cutoff of minimum 96% identity over 92% consecutive nucleotides of a read. Those uniquely aligned reads (reads mapped to unique locations in the reference genome) were retained. These reads were used to call the single-base pair genotypes of the consensus sequences across the whole genome using the Ssaha_Pileup package (v0.5). Among them, the low-quality bases (that is, base-quality Q score in Phred scale < 25) were removed, and those sites with conflicting genotypes among different reads were also excluded. Moreover, the overall depth in each site is required to be less than 100 to avoid mapping to regions with copy number variation. In the genome sequences of the two parents, 1,163,670 SNPs were identified between them and used as potential markers for genotyping.

Genomes of the CSSLs were re-sequenced on the Illumina Genome Analyzer IIx using the multiplexed sequencing and paired-end strategy. Purified genomic DNA of each CSSL individual was sheared by sonication (Bioruptor XL™, diagenode) to fragments of less than 800 bp. End repair of the fragments was performed by treating them with T4 DNA polymerase, T4 polynucleotide kinase and Klenow DNA polymerase. The obtained blunt phosphorylated DNA fragments were treated with Klenow fragment (3' to 5' exo minus) and dATP to generate a protruding 3' A, and ligated to paired-end adaptors carrying a four-base indexing tag, as proposed by Cronn et al. [46]. The fourth base of the index was a thymidine used for ligation to the 3' A overhang DNA fragments. All sixty-four possible indices were used as a tag for adaptors. Approximately 26-28 indexed DNAs from CSSLs of an equimolar concentration were pooled and loaded on to 2% agarose gels. Fragments of 300-400 bp were recovered and purified and enriched by 18 cycles of PCR to obtain a solexa sequencing library. Each library was loaded on to one lane of the Illumina GAIIx for 2 × 40 bp paired-end sequencing, with the Illumina PhiX sample used as a control. Image analysis and base calling were performed using Illumina GAPipeline v1.4.0. Therefore, four-base indexed DNAs of 26-28 CSSLs were combined and sequenced in a single lane, and complete sequencing of 128 CSSLs was performed in a single run.

Sequences were sorted and aligned with the pseudo-molecules of the parental genome sequences for SNP detection. For each CSSL, the reads of 40-bp sequences (36-mers excluding the index) were sorted according to the 5' indexes. Approximately 50 Mb sequences were generated for each CSSL, which was equivalent to 0.13× coverage of the rice genome. Detected SNPs were arranged along the chromosomes according to their physical locations with their genotypes indicated. A sliding window approach was used for genotype calling, recombination breakpoint determination and map construction based on the detected SNPs [47]. Background noise due to sequence errors and mapping errors precluded determination of the genotype of CSSLs on the basis of individual SNPs alone. In order to identify the genotype for each genomic region accurately and identify recombination breakpoints precisely, a Perl script, *Seq2bin*, was adopted http://www.ncgr.ac.cn/software/SEG. This uses a sliding window approach to evaluate a group of consecutive SNPs for genotyping, and the window size was dynamic according to the SNP density of the CSSL.

### Phenotyping for culm length in CSSLs

In total, 128 CSSLs, 9311 and Nipponbare were grown in the experimental farm of Yangzhou University, Yangzhou (33°N, 119°E), China, under natural conditions, in the summer of 2009. A randomized complete block design with one replication was used to lay out the trial. Each plot consisted of forty plants in four rows. Ten plants in the center of each plot were selected to investigate their characteristics.

### CSSLs-based QTL mapping

**Figure 7:**
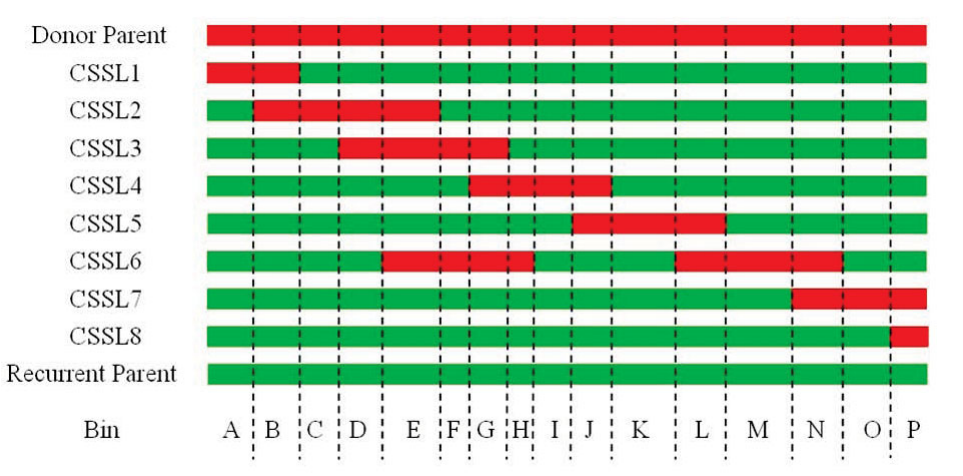
**A hypothetical CSSL library with eight CSSLs, a donor parent and a recurrent parent**. The red areas indicate regions that are homozygous for donor alleles; the green areas indicate regions homozygous for recurrent alleles.

Figure 7 shows a hypothetical CSSL library, in which each line contains a single or several donor segments (red bars). There was a little overlap of the donor segments between or among different lines. QTL mapping with high resolution benefits from this overlapping. To locate the QTLs precisely in terms of the donor segments, the overlapping portions of the CSSLs were utilized to delineate smaller segment sizes. These smaller segments were called bins [48]. The commonly-used bin mapping scheme was achieved by phenotyping each of the lines in randomized replicated trials and presenting the results as the difference from the recurrent parent. The bins with significant contribution could be detected [49,50]. In this study, each bin was taken as an independent variable. For instance, bins from A to P in Figure 7 were defined as *x*_1 _to *x*_16_. Therefore, the main effect model was described by the following multiple linear model, where *y_i _*is the mean value of the *i*th line of a CSSLs library, *b*_0 _is the overall mean of the population, *m *is total number of bins in the entire genome, *b_k _*is the main effect associated with bin *k*. *x_ik _*is an indicator variable, denoting *x_ik _*= 1 for donor parent bin, *x_ik _*= -1 for recurrent parent bin. *e_j _*denotes the residual error following a normal distribution:

$$y_{i}=b_{0}+\sum_{k=1}^{m}b_{k}x_{ik}+e_{i}$$

Contributions of the target bins to phenotypic variation were estimated using a multiple linear regression analysis with the stepwise option in the REG procedure of the SAS software package (SAS Institute Inc. 2000). In the stepwise method, variables are added one by one to the model and the F statistic for a variable to be added must be significant at the SLENTRY = level. However, after a variable is added, the stepwise method considers all the variables already included in the model and deletes any variable that does not produce an F that is statistically significant at the SLSTAY = level. Only after this check is carried out and the necessary deletions are accomplished can another variable be added to the model. The stepwise process ends when none of the variables outside the model has an F statistically significant at the SLENTRY = level and every variable in the model is significant at the SLSTAY = level, or when the variable to be added to the model is the one just deleted from it.

In our analysis, the explanatory variables included the main effects of these target regions. The significance level was fixed at 0.01 for both SLENTRY and SLSTAY. The contribution of each significant explanatory variable to the dependent variable was displayed with the coefficient of determination that was calculated by the corresponding partial regression sum of squares divided by the sum of squares of the dependent variable.

## Authors' contributions

JJX performed the majority of the experiments and wrote the paper. QZ was responsible for the whole-genome re-sequencing. CWX was responsible for QTL mapping. PND, BHW, QF, QQL and SZT each performed several experiments. MHG provided valuable suggestions for the project. BH designed the whole-genome re-sequencing experiment. GHL designed the other experiments. All authors read and approved the final manuscript.
